# Unveiling Histiocytic Sarcoma: A Rare Case of Diagnostic Complexity and Therapeutic Resistance

**DOI:** 10.7759/cureus.80927

**Published:** 2025-03-20

**Authors:** Abid Nawaz Khan Adil, Bakht Rehman, Syed S Raza, Muhammad A Khan

**Affiliations:** 1 Internal Medicine, Community Medical Center, Fresno, USA; 2 Internal Medicine, Khyber Medical College/Teaching Hospital, Peshawar, PAK; 3 Internal Medicine, University of Kansas Medical Center, Kansas City, USA

**Keywords:** complexity of diagnostic challenges, histiocytic sarcoma, immune checkpoint inhibitors, immunohistochemistry and biopsy, therapeutic resistance

## Abstract

Histiocytic sarcoma (HS) is a rare and aggressive hematological malignancy. It is derived from the lineage of macrophages or monocytes. HS commonly presents as a mass in areas such as the gastrointestinal tract and soft tissues. This case highlights the clinical challenges in diagnosing and managing HS in a 37-year-old female patient. Accurate diagnosis relies on immunohistochemistry and molecular studies, while treatment outcomes remain suboptimal. The patient underwent multimodal therapies, including chemotherapy, targeted therapy, and radiation therapy, with disease progression observed despite treatment. This report underscores the need for novel approaches to improve outcomes in HS and the role of ARID2 mutations, which in our case limits the applicability of immune checkpoint inhibitors in disease management.

## Introduction

Histiocytic sarcoma (HS) is an extremely rare occurrence, with less than 1% of hematopoietic tumors originating from histiocytes [1,2]. Characterized by aggressive clinical behavior, HS often mimics other lymphoid malignancies, posing diagnostic challenges. HS has a mean incidence age of 53 years with an age range of 15-89 years. It typically presents as a mass with an average size of 6 cm, ranging from 1.8 cm to 12 cm. Common sites include the gastrointestinal tract and soft tissues. Hematoxylin and eosin (H&E) staining reveals cells having eosinophilic cytoplasm and an irregular nucleus [3]. Accurate diagnosis relies on immunohistochemistry (IHC) and molecular studies, while treatment outcomes remain suboptimal [1,4].

Given the rarity and histologic overlap with diverse mimics, the diagnosis of HS can be extremely challenging. Thus, recognizing morphologic clues, as well as judicious application of IHC markers for confirming the histiocytic lineage and to exclude mimics, is pivotal for the diagnosis [1]. This case highlights the clinical challenges in diagnosing and managing HS in a 37-year-old female patient. It is expected that our study results will provide a novel perspective on the management of HS.

## Case presentation

A 37-year-old female patient presented to her family physician with complaints of persistent right-sided chest pain radiating to the back, low-grade fever, gastroesophageal reflux symptoms, a significant weight loss of 60 lbs over one year, and night sweats. Her past medical history was significant for seasonal allergies, well-controlled asthma, and migraine headaches.

An upper gastrointestinal endoscopy was planned which showed mild gastritis. The laboratory tests are tabulated in Table 1.

**Table 1:** Laboratory investigations

Test	Observed value	Reference range
Hemoglobin	8.4 g/dL	12.1-15.1 g/dL
Platelets	589×10^9^/L	150-450×10^9^/L
Iron saturation	<4%	20-50% (males)
C-reactive protein	109 mg/L	<10 mg/L

**Figure 1:**
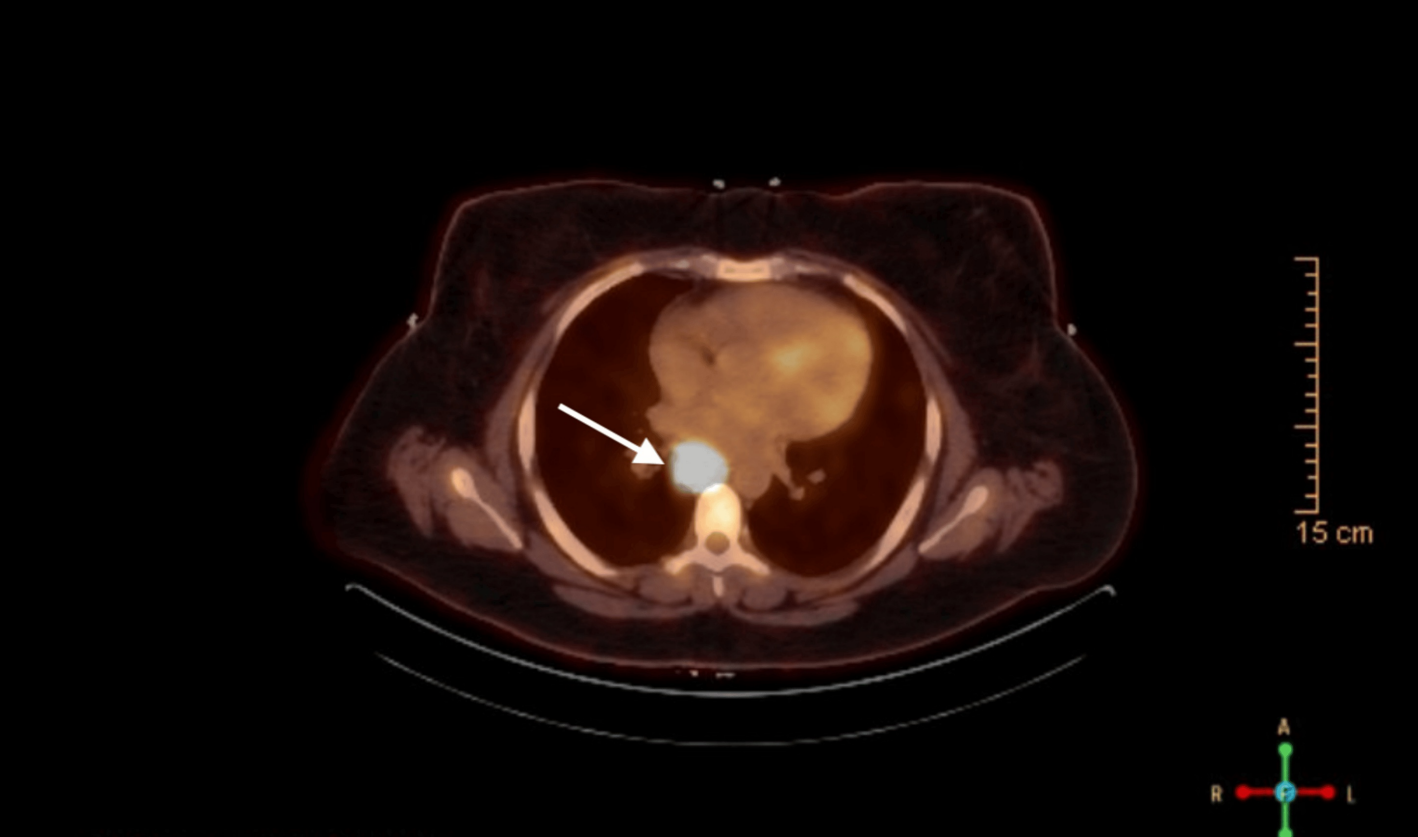
PET-CT scan demonstrating FDG-avid subcarinal lymph nodes PET-CT: positron emission tomography-computed tomography; FDG-avid: fluorodeoxyglucose-avid

A contrast-enhanced computed tomography (CT) scan identified anterior mediastinal lymphadenopathy. Positron emission tomography (PET)-CT demonstrated fluorodeoxyglucose (FDG) uptake showing splenic involvement and subcarinal lymph nodes (maximum standardized uptake value (SUVmax) 27), suggestive of malignancy (Figure 1).

The image shows hypermetabolic activity in the subcarinal lymph nodes with a SUVmax of 27. FDG uptake is consistent with increased metabolic activity, indicative of potential malignancy. Additionally, splenic involvement and mesenteric lymphadenopathy can be noted in the image.

**Figure 2:**
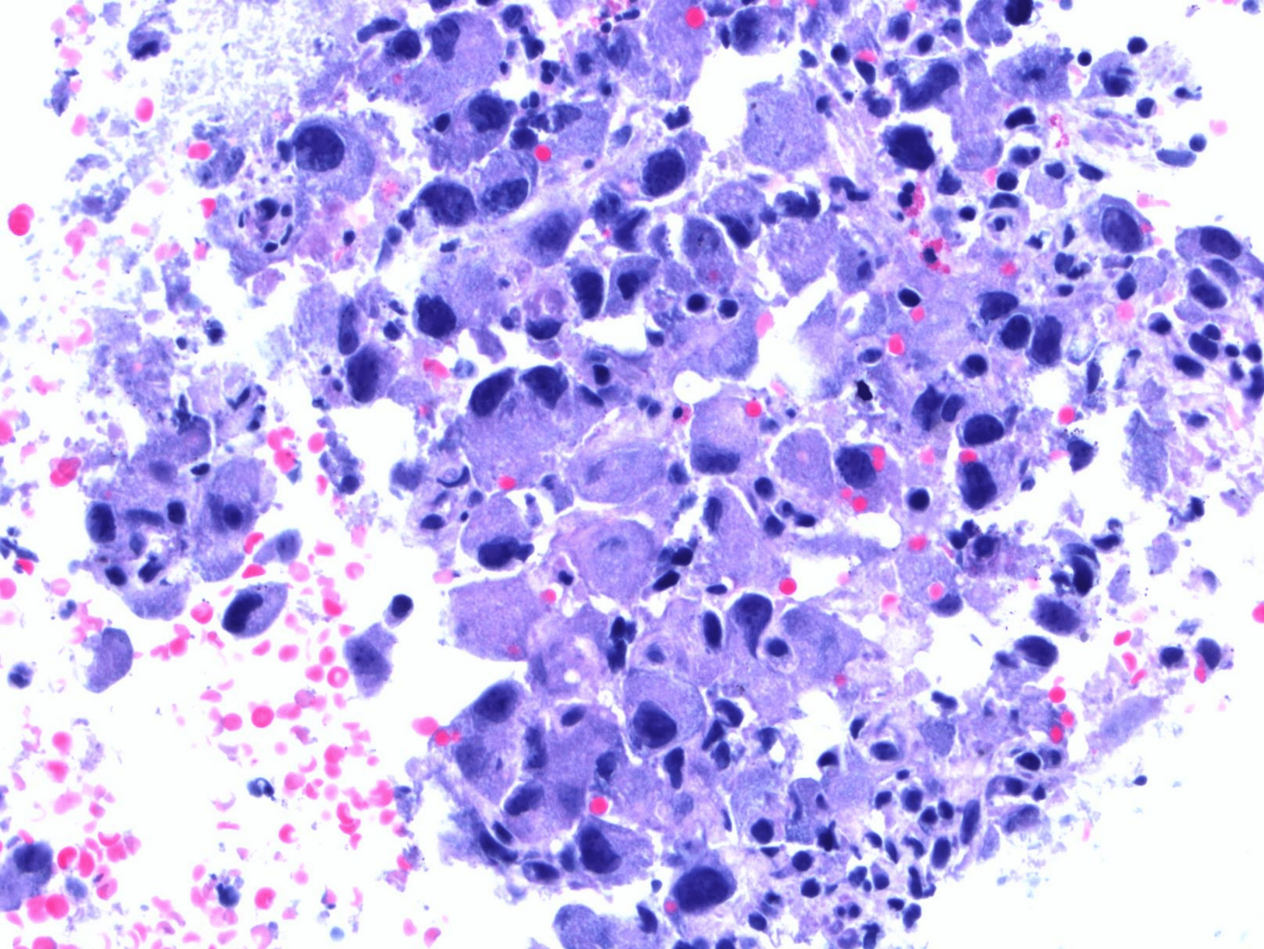
H&E at 400× showing large cells with round to irregular hyperchromatic and eccentric nuclei, occasional multinucleated forms, and abundant amphophilic cytoplasm confirming histiocytic sarcoma H&E: hematoxylin and eosin

Bone marrow biopsy revealed a normocellular (60-70%) marrow and mild reticulin fibrosis without any significant lymphoid immunophenotypic abnormality. This was followed up by endobronchial ultrasound (EBUS) and biopsy, which initially hinted at poorly differentiated anaplastic T-cell lymphoma (ALK-negative and CD30-positive). A second opinion at the National Institutes of Health (NIH) confirmed HS as shown in Figure 2.

**Figure 3:**
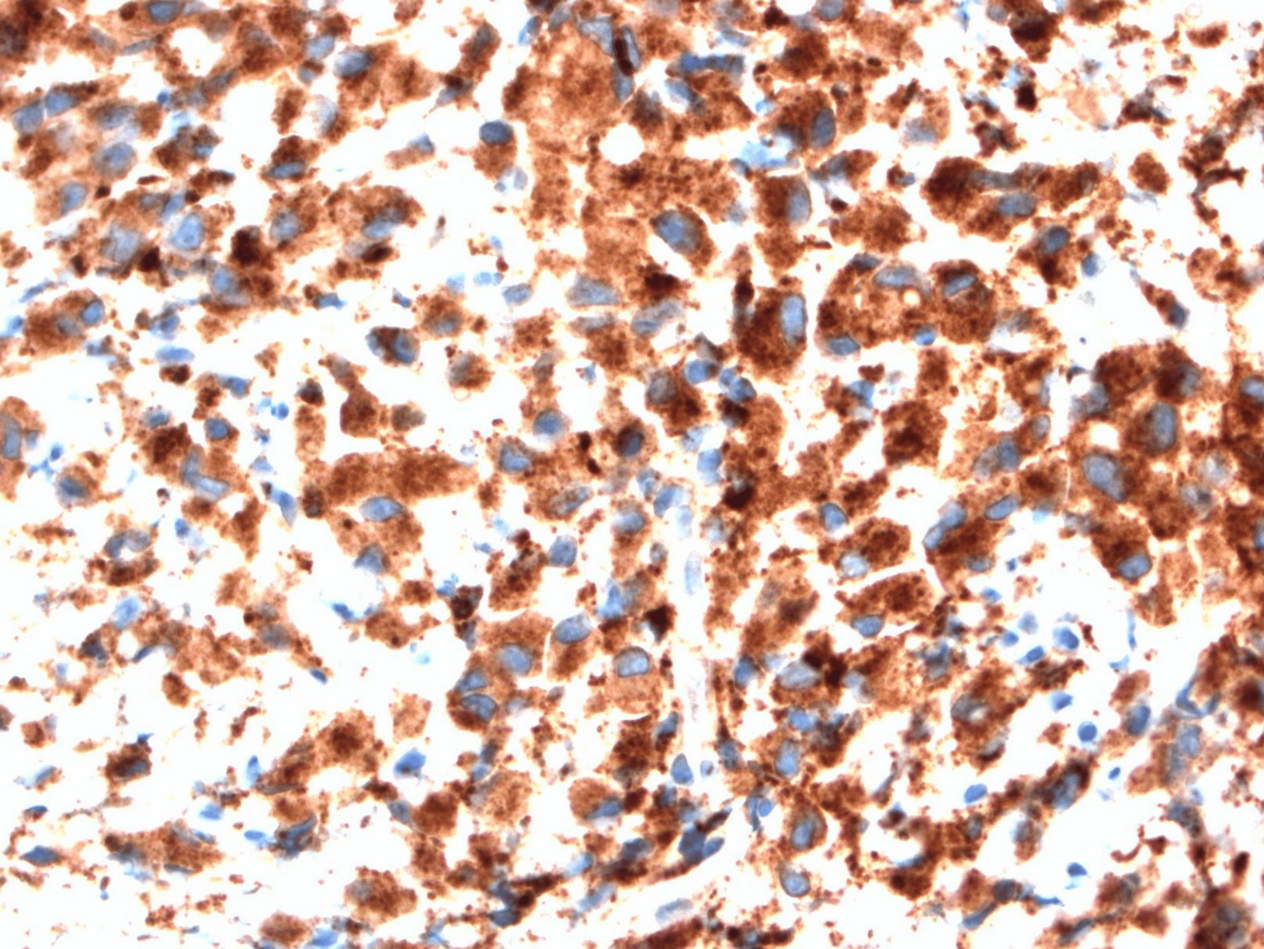
CD68 at 400× showing strong cytoplasmic staining to support histiocytic differentiation

IHC was positive for cyclin D1, CD30, CD68, C-MYC, EMA1, and vimentin and negative for CD2, CD4, CD5, CD8, CD15, CD20, PAX5, ALK, granzyme, perforin, LCA, MUM1, and Epstein-Barr virus (Figure 3).

Treatment and progression

First-Line Therapy: A+CHP

**Figure 4:**
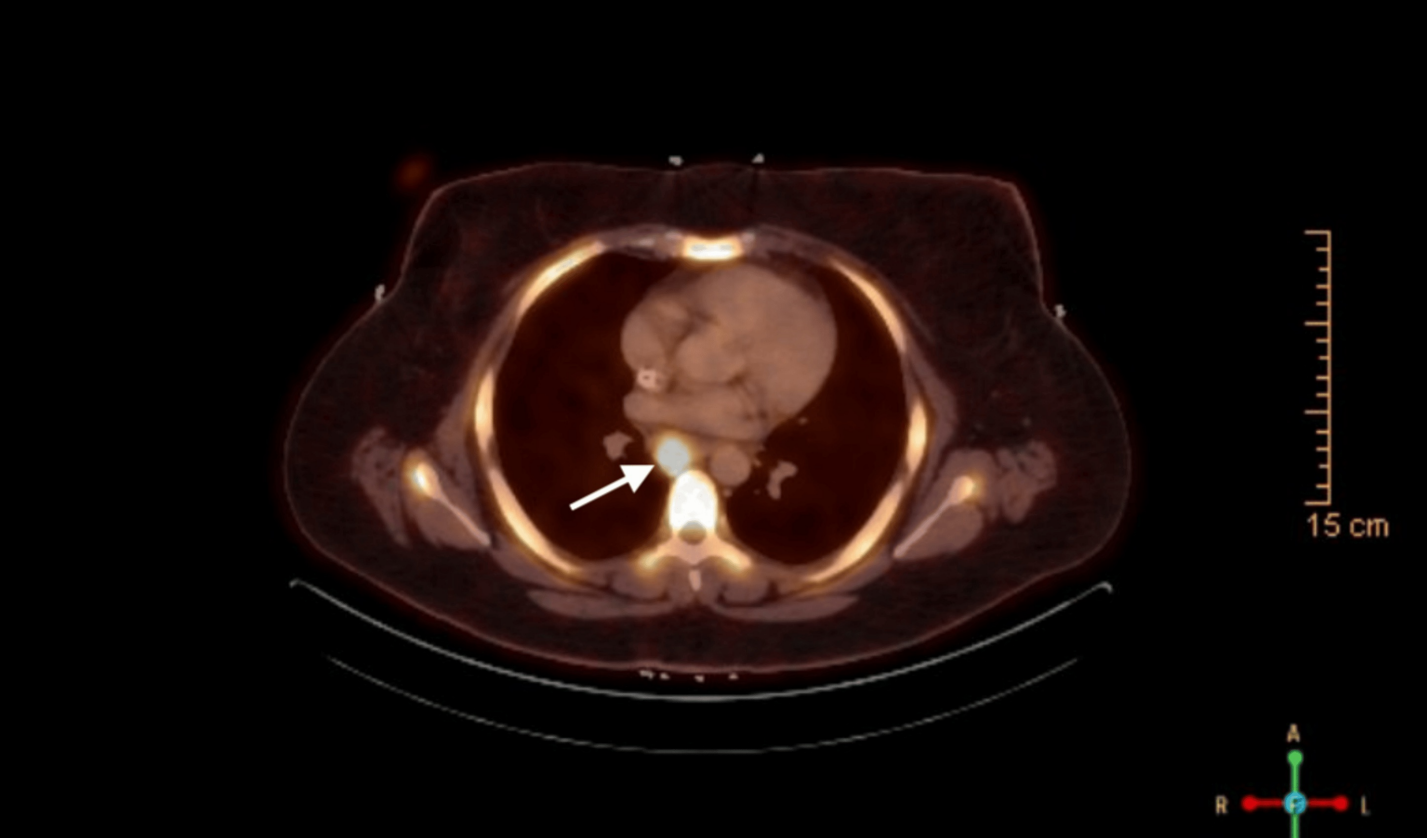
PET-CT scan demonstrating suboptimal response to therapy The PET-CT scan from January 2024 shows persistent FDG uptake in the mediastinal lymph nodes, with a reduced SUVmax of 19 in comparison to the baseline SUVmax of 27. The reduction in lymph node size is limited to <30%. This indicates a suboptimal therapeutic response. These findings suggest residual disease activity and inadequate metabolic response to the administered chemotherapy regimen. PET-CT: positron emission tomography-computed tomography; FDG: fluorodeoxyglucose; SUVmax: maximum standardized uptake value

In November 2023, the patient was started on the A+CHP chemotherapy (brentuximab vedotin, cyclophosphamide, doxorubicin, and prednisone) and underwent three cycles. This was followed by a PET-CT, which showed persistent FDG activity with little reduction in lymph node size (<30%), indicating a suboptimal response (Figure 4) [3,5].

Second-Line Therapy

In February 2024, the regimen was switched to ICE (ifosfamide, carboplatin, and etoposide). PET-CT following two cycles showed a partial response [3,6].

Targeted Therapy

Cobimetinib, a MEK inhibitor, was administered for four cycles but discontinued due to severe diarrhea. Trametinib was later introduced to improve responses before radiation therapy.

Radiation Therapy

The patient received stereotactic body radiation therapy (SBRT) targeting mediastinal lymphadenopathy. A total dose of 54-60 Gy was planned to be given split over 27-30 sessions. However, a PET-CT in December 2024 showed new FDG uptakes in the paratracheal lymph nodes and retropharyngeal region with evidence of bone metastasis, indicating disease progression.

Future plans

Allogeneic transplantation is being considered in this patient. While transplantation offers a potential therapeutic option, its success is contingent on achieving a better response to standard therapy [7,8].

## Discussion

HS presents significant diagnostic and therapeutic challenges due to its rarity and aggressive behavior. Accurate diagnosis requires detailed IHC and molecular profiling [1,3,4]. HS is a rare and aggressive hematologic malignancy with median survivals ranging between six and 16 months [9].

Diagnosing HS is very challenging. There is a range of differentials including but not limited to non-Hodgkin lymphomas, poorly differentiated carcinomas, or sarcomas. Immunohistochemical staining confirms the diagnosis. It must be positive with at least one of the following: CD68, CD163, lysozyme, or 11c. On rare occasions, CD15 or CD30 staining may be weakly positive [10].

The lack of standardized treatment protocols in HS demands a multimodal approach. Furthermore, this case demonstrates the limited efficacy of the existing management therapies. Targeted agents, chemotherapy, and radiation therapy have limited efficacy in achieving sustained remission [4,5]. Treatment usually involves a multimodal approach including chemotherapy, targeted therapy, radiation therapy, and surgery. Traditional chemotherapy with CHOP (cyclophosphamide, doxorubicin, vincristine, and prednisone) followed by etoposide has been used and shown regression in some cases. This is followed by high-dose chemotherapy and autologous transplantation on a case-to-case basis. Similarly targeted therapies for patients based on molecular profiling have shown promise [11].

This patient's tumor revealed ARID2 mutations with microsatellite stability, which limits the applicability of immune checkpoint inhibitors in disease management [1]. ARID2 gene mutations are not commonly associated with the RAS/RAF/MAPK and PI3K/AKT pathways in the context of HS. The literature on HS primarily highlights mutations in the RAS/RAF/MAPK and PI3K/AKT pathways, with frequent alterations in genes such as KRAS, NRAS, BRAF, MAP2K1, NF1, PTEN, MTOR, PIK3R1, and PIK3CA. These pathways are critical in the pathogenesis of HS, driving cell proliferation and survival, while ARID2 is involved in the SWI/SNF chromatin remodeling complex, leading to altered gene expression. This disruption may affect the regulation of key genes within the RAS/RAF/MAPK and PI3K/AKT pathways, resulting in aberrant cell growth. The resulting dysregulation of these signaling pathways can contribute to tumorigenesis and cancer progression. This has been implicated in various cancers, but its specific role and prevalence in HS have not been well-documented. Therefore, ARID2 mutations do not appear to play a significant role in the RAS/RAF/MAPK and PI3K/AKT pathways in HS [12]. Furthermore, our patient had a frameshift mutation of ARID2 which has not been directly involved in HS but has been documented in other cancers.

## Conclusions

This case underscores the urgent need for superior and improved diagnostic tools along with better therapeutic strategies in managing HS. The currently available therapies provide temporary relief with disease control. However, the long-term prognosis remains poor. Further studies aimed at the molecular pathogenesis of HS may help develop novel and effective therapeutic targets that could potentially improve survival. This report also highlights the role of ARID2 mutations, which in our case limits the applicability of immune checkpoint inhibitors in disease management.
